# Acute kidney injury in lamotrigine-induced DRESS syndrome

**DOI:** 10.1007/s00467-024-06397-3

**Published:** 2024-05-27

**Authors:** Klara Kristin Brüning, Elena Pelivan, Marie-Christine Heinrich, Philip Bufler, Angela Kaindl, Julia Thumfart

**Affiliations:** 1 Klinik Für Pädiatrie m. S. Gastroenterologie, Nephrologie Und Stoffwechselmedizin, Charité Universitätsklinikum, Berlin, Germany; 2 Klinik Für Pädiatrie m. S. Neurologie, Charité Universitätsklinikum, Berlin, Germany; 3 Institut Für Pathologie, Charité Universitätsklinikum, Berlin, Germany

**Keywords:** DRESS (drug reaction with eosinophilia and systemic symptoms), Lamotrigine, Acute interstitial nephritis (AIN), Non-adherence

## Abstract

We present a case of lamotrigine-triggered DRESS (drug reaction with eosinophilia and systemic symptoms) syndrome with acute kidney injury stage 3. A 17-year-old girl with known epilepsy treated with lamotrigine presented with acute kidney injury as well as skin eruption, fever, and apathy. Extended diagnostics, considering infectious and autoimmune diseases, remained unremarkable. Lamotrigine blood levels were within the target range. Kidney biopsy showed acute interstitial nephritis with tubular necrosis. Methylprednisolone pulse therapy led to an improvement in kidney function; skin eruption and neurological symptoms resolved. During the hospital stay, the girl admitted to inconsistent and variable intake of lamotrigine, occasionally resulting in notable overdosing. This report demonstrates that acute kidney injury in lamotrigine-induced DRESS syndrome is an acute interstitial nephritis with tubular necrosis, an aspect that has not been deeply characterized so far. Additionally, we aim to elevate awareness towards non-adherence as cause of disease, especially among the adolescent population.

## Introduction

DRESS (drug reaction with eosinophilia and systemic symptoms) or HSS (anti-convulsant hypersensitivity syndrome) describe a rare, heterogeneous clinical entity of severe adverse drug reaction. Even though the syndrome’s existence is broadly accepted, diverse symptoms make clear diagnostic criteria hard to establish. DRESS therefore remains a diagnosis of exclusion. In 2007, the European Registry of Severe Cutaneous Adverse Reaction Criteria (RegiSCAR) study group developed inclusion criteria as well as a scoring system classifying suspected cases as no, possible, probable or definite DRESS case [1]. RegiSCAR inclusion criteria are: hospitalization, reaction suspected to be drug related, and at least three criteria of acute skin rash, fever above 38°, enlarged lymph nodes, involvement of at least one internal organ and blood count abnormalities (e.g. lymphocytes above or below laboratory limits). Yet indicating otherwise, DRESS syndrome does frequently lack eosinophilia and is not part of currently used diagnostic criteria. Drugs at high risk of causing DRESS are especially aromatic anticonvulsants (e.g. carbamazepine, phenytoin, lamotrigine), allopurinol and antibiotics, such as beta-lactams and vancomycin [1]. Latency period between initiation of medication and appearance of symptoms varies between 1 and 8 weeks.

## Case report

**Fig. 1:**
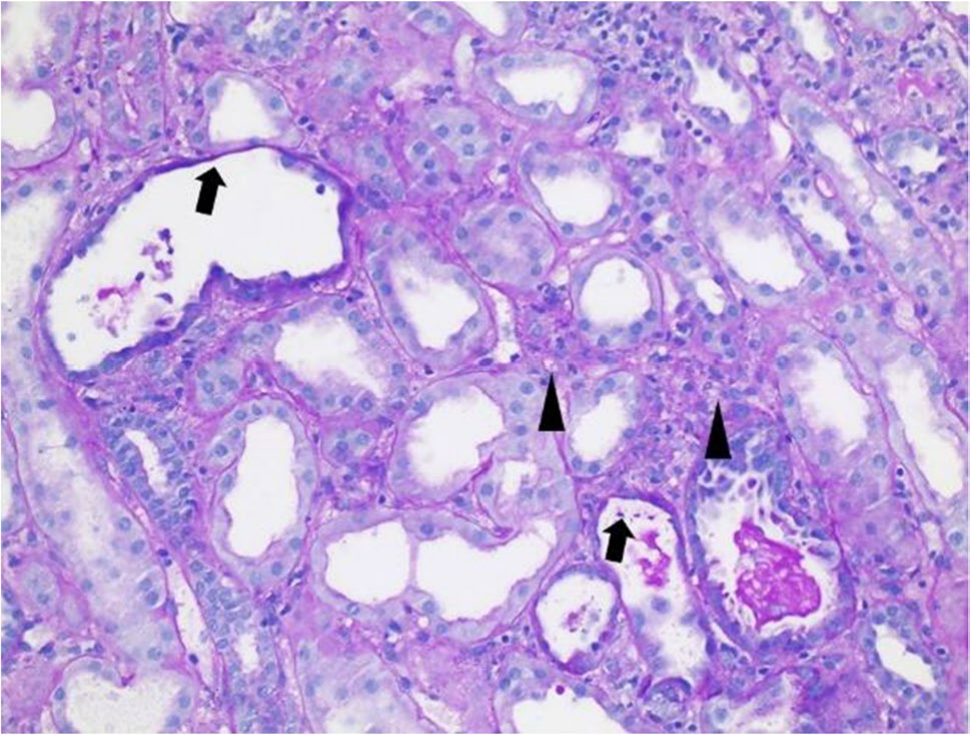
Kidney biopsy of a patient with lamotrigine-induced DRESS showing acute interstitial nephritis (AIN) (▲) and acute tubular necrosis (ATN) (  )

A 17-year-old girl presented in the emergency department in impaired clinical condition with fever up to 40 °C, apathy, and a generalized maculopapular exanthema without affection of the mucosa. Lymph nodes were not enlarged. Blood samples showed hyponatremia of 129 mmol/l, increased levels of creatinine of 3.1 mg/dl (estimated glomerular filtration rate 21 ml/min/1.73 m^2^) and C-reactive protein of 51 mg/l, reduced hemoglobin of 8.2 g/dl as well as lymphopenia of 0.55/nl. Due to suspected meningitis, our patient received treatment with cefotaxime and acyclovir, which was discontinued when normal results from cMRI and cerebrospinal fluid analysis were obtained. Serum creatinine rose to 7 mg/dl and the patient developed slight pericardial effusion and general edema. The upper skin layers of the fingers were peeling. Transient QT-prolongation normalized over time. The patient was treated with furosemide and fluid intake restriction. Diuresis was continuously preserved. Regarding differential diagnosis ParvoB19, CMV, EBV, HHV6 and 8, (para)influenza, Adeno-, Entero- or Hanta-Virus were ruled out by negative serology or PCR. Ophthalmology consultation did not show evidence of inflammatory disease. Blood culture remained negative. Echocardiography did not show signs of endocarditis. Autoimmune parameters such as ANA, p- and c-ANCA, anti-dsDNA, antiphospholipid antibodies and anti-glomerular basement membrane antibodies were normal. Slight proteinuria of alpha1-microglobulin and albumin was detected (max. 280 mg/g protein/creatinine ratio). Kidney biopsy showed acute interstitial nephritis (AIN) as well as acute tubular necrosis (ATN) (see Fig. 1). A methylprednisolone pulse (500 mg/m^2^ body surface area) was administered for 3 days and followed by oral prednisolone.

The girl had an epilepsy of unknown etiology with generalized and focal onset tonic–clonic seizures and had been treated with lamotrigine and clobazam for the past 8 months. Genetic and metabolic analysis had not revealed etiology of the epilepsy. She experienced her last tonic–clonic seizure 1 month prior to admission resulting in an adjustment of lamotrigine dosage from 175 to 200 mg daily due to low blood levels.

Initially, the patient denied potential overdosing and drug levels were within the target range. Several days after hospital admittance, the patient admitted to lamotrigine non-adherence — omitting the medication on some days and taking multiple tablets on others. She stated the fear of weight gain, being a known side effect of lamotrigine, as one reason. Subsequently, we temporarily discontinued the anti-seizure medication.

Creatinine and edema decreased; the general condition improved. The patient was discharged 2 weeks after admission and exhibited further enhancement in kidney function. However, complete recovery remains unattained even 5 months post-discharge (68 ml/min/1.73 m^2^). Anti-seizure medication was transitioned from lamotrigine to levetiracetam, causing less severe side effects in case of overdose.

## Discussion

Recognizing lamotrigine as a known trigger of cutaneous adverse events, we identified DRESS syndrome as the best fitting diagnosis. Our patient met the RegiSCAR inclusion criteria, and the corresponding scoring system classified the case as a probable manifestation of DRESS [1]. After comprehensive exclusion of differential diagnoses, we were therefore able to confirm the diagnosis.

The prevalence of kidney involvement in DRESS-syndrome differs considerably, ranging between 13 and 35% of cases with internal organ damage [2]. Kidney failure in DRESS syndrome is generally due to AIN (classic triad: rash, eosinophilia, fever) [2], yet the precise etiology remains uncertain. The suspected pathomechanism of drug-induced AIN and, interestingly, also DRESS, involves an idiosyncratic delayed type IV hypersensitivity reaction. It is initiated by the formation of haptens, which provoke a cellular immune response, either through direct cellular damage or through the release and deposition of antibodies [3]. DRESS and AIN have previously been described as distinct clinical entities, despite their shared symptoms such as fever, rash, and a suspected common etiology. We therefore propose to establish a common definition for a drug-induced syndrome involving kidney damage, ideally based on its etiology.

In our case, histological examination revealed interstitial nephritis along with tubular necrosis, confirming severe AIN with a tubular necrotic component, consistent with findings in other DRESS cases with renal involvement [2], (see Fig. 1).

AIN and DRESS, in contrast to ATN, are considered to be dose-independent [2]. However, in our patient we suspect that non-adherence to prescribed doses of antiepileptic drugs, resulting in inconsistent and temporarily elevated drug levels, contributed to kidney failure. For lamotrigine, the risk of rash has been reported to increase if the initial dose or dose escalation rate is exceeded. Here, both applied [4].

In the absence of controlled studies, treatment recommendations for DRESS are based on empirical and consensus-based approaches using mainly systemic corticosteroids tapered over 3 months. Long-term prognosis has not been reported systematically.

The initially normal drug levels of lamotrigine were misleading in the diagnostic process. This report aims to raise awareness of the prevalent risks of treatment non-adherence in the pediatric population. Approximately 50% of children are reported not to adhere to treatment regimens, a number that tends to increase in adolescence [5].

## Conclusion

We propose that overdosing of lamotrigine induces acute kidney injury by AIN combined with ATN as part of DRESS or HSS. When assessing drug-induced side effects in pediatric patients, non-compliance might be present even if drug levels are within the normal range.

## Summary

### What is new?

- Lamotrigine-induced DRESS syndrome can cause acute kidney injury due to acute interstitial nephritis (AIN) and acute tubular necrosis (ATN).
- A single drug target level should not lead to exclusion of drug-associated disease etiology.

## Data Availability

All data generated or analysed during this study are included in this published article.
